# Identification of a unique Radical SAM methyltransferase required for the sp^3^-C-methylation of an arginine residue of methyl-coenzyme M reductase

**DOI:** 10.1038/s41598-018-25716-x

**Published:** 2018-05-09

**Authors:** Darja Deobald, Lorenz Adrian, Christian Schöne, Michael Rother, Gunhild Layer

**Affiliations:** 1 Leipzig University, Institute of Biochemistry, Brüderstraße 34, 04103 Leipzig, Germany; 2 Helmholtz Centre for Environmental Research – UFZ, Isotope Biogeochemistry, Permoserstraße 15, 04318 Leipzig, Germany; 3 Technische Universität Berlin, Chair of Geobiotechnology, Ackerstraße 74, 13355 Berlin, Germany; 4 Technische Universität Dresden, Institute of Microbiology, Zellescher Weg 20b, 01217 Dresden, Germany

## Abstract

The biological formation of methane (methanogenesis) is a globally important process, which is exploited in biogas technology, but also contributes to global warming through the release of a potent greenhouse gas into the atmosphere. The last and methane-releasing step of methanogenesis is catalysed by the enzyme methyl-coenzyme M reductase (MCR), which carries several exceptional posttranslational amino acid modifications. Among these, a 5-C-(*S*)-methylarginine is located close to the active site of the enzyme. Here, we show that a unique Radical *S*-adenosyl-L-methionine (SAM) methyltransferase is required for the methylation of the arginine residue. The gene encoding the methyltransferase is currently annotated as “methanogenesis marker 10” whose function was unknown until now. The deletion of the methyltransferase gene *ma4551* in *Methanosarcina acetivorans* WWM1 leads to the production of an active MCR lacking the C-5-methylation of the respective arginine residue. The growth behaviour of the corresponding *M*. *acetivorans* mutant strain and the biophysical characterization of the isolated MCR indicate that the methylated arginine is important for MCR stability under stress conditions.

## Introduction

The biological formation of methane represents an important branch of the global biogeochemical carbon cycle. Each year, about one billion tons of methane are produced by methanogenic archaea with about one third of the amount escaping into the atmosphere^1^. Although methane produced in biogas plants serves for energy generation, it also acts as a potent greenhouse gas whose increased emission contributes to global warming. Methanogenic archaea conduct methanogenesis as their sole strategy of energy conservation by using CO_2_ and H_2_, acetate or several C1-compounds such as methanol or methylamines as their substrates for methane formation^2,3^. Although the methanogenic pathways differ for these substrates, they all share the last and methane-releasing step, which is catalysed by methyl-coenzyme M reductase (MCR)^3–5^. Accordingly, the encoding genes (*mcrA*, *mcrB*, *mcrG*) are found in the genomes of all methanogenic archaea. Besides its essential role in methanogenesis, MCR also catalyses the initial step of anaerobic oxidation of methane (AOM) performed by anaerobic methanotrophic archaea (ANME)^6–9^.

**Figure 1:**
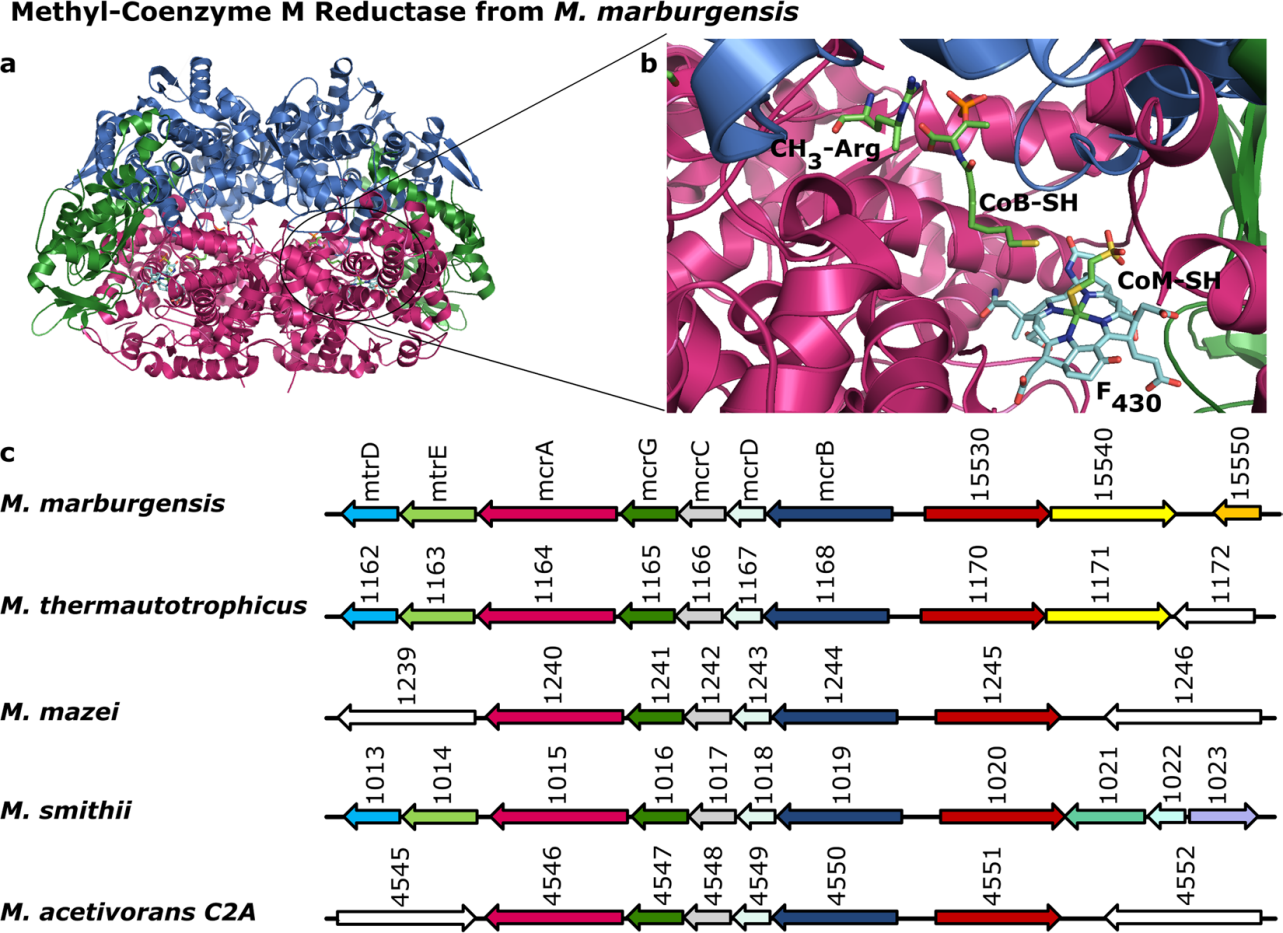
Crystal structure of methyl-coenzyme M reductase from *M*. *marburgensis* and the organisation of the *mcr* operon. (**a**) The crystal structure of methyl-coenzyme M reductase from *M*. *marburgensis* is shown in cartoon representation with the McrA (α) subunit in magenta, the McrB (β) subunit in marine and the McrG (γ) subunit in green. (**b**) View into the active site and the substrate channel with the bound coenzyme F_430_ (turquoise), CoB-SH and CoM-SH as well as the modified amino acid residue 5-methylarginine (all green) in stick representation. The colour code of the protein subunits is the same as in (**a**). (**c**) Comparison of the genome regions neighbouring the *mcr* operon of selected methanogenic archaea with the colour code for *mcrA*, *mcrB* and *mcrG* as in (**a** and **b**). Most methanogens possess the gene for a predicted Radical SAM enzyme (red), usually annotated as “methanogenesis marker 10”, located next to *mcrB*. Furthermore, some methanogenic archaea carry a gene encoding a putative cobalamin-dependent Radical SAM enzyme (yellow).

MCR consists of the subunits McrA (α), McrB (β) and McrG (γ), which form a dimer of heterotrimers (αβγ)_2_ (Fig. 1a)^10,11^. The enzyme catalyses the reversible reduction of methyl-coenzyme M (CH_3_-S-CoM) together with the co-substrate coenzyme B (CoB-SH) to methane under concomitant formation of the heterodisulfide CoB-S-S-CoM^5,12^. The reaction most likely proceeds *via* a methyl radical intermediate and relies on coenzyme F_430_ exclusively found in MCR^4,13^. Another exceptional trait of MCR are several unusual posttranslational amino acid modifications located near the active site. So far, in methanogenic MCR the following modifications were identified within the McrA subunit: 1-*N*-methylhistidine, *S*-methylcysteine, 5-C-(*S*)-methylarginine (Fig. 1b), 2-C-(*S*)-methylglutamine, thioglycine, didehydroaspartate and 6-hydroxy-tryptophan^11,14–18^. Depending on the organism and specific MCR isoenzyme, not always all of these modifications are present. Moreover, the α-subunit of the MCR from an ANME-1 methanotroph possesses a 7-hydroxy-tryptophan and does not carry the 5-methylarginine^14,19^.

**Figure 2:**
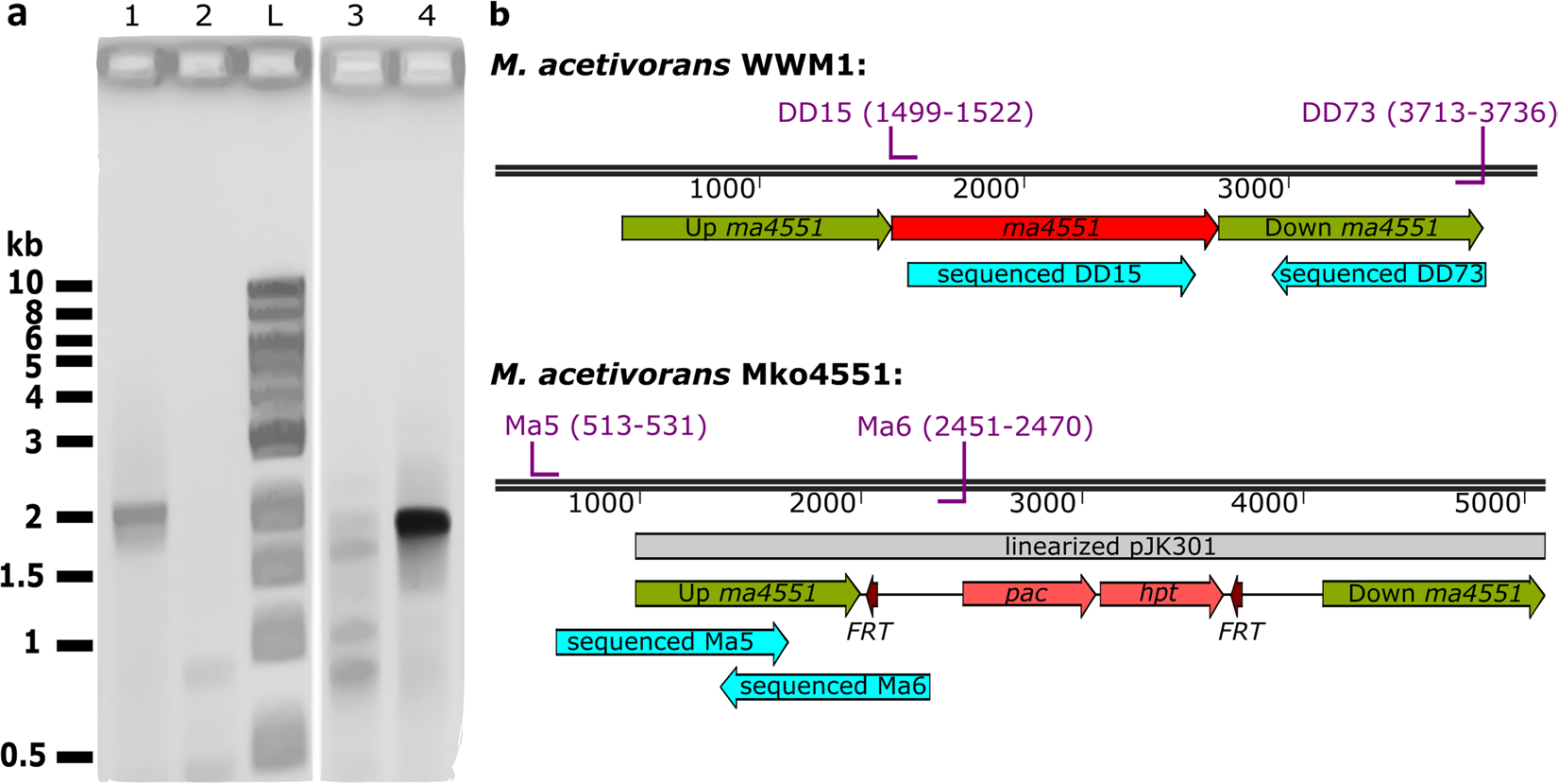
PCR based illustration of the *M*. *acetivorans* WWM1 and Mko4551 genotypes. (**a**) Verification of genotypes by PCR. Amplification of the *ma4551* region using primers DD15 and DD73 from wild type DNA yielded a 2.2 kb fragment (lane 1), which was not obtained from Mko4551 DNA (lane 2). A part of the *pac-hpt* cassette was successfully amplified from Mko4551 DNA using primers Ma5 and Ma6 (lane 4) (ca. 2 kb), but not from wild type DNA (lane 3). Lane L: Quick-Load® 1 kb DNA Ladder (New England BioLabs). (**b**) Genotype of the *M*. *acetivorans* WWM1 and Mko4551 with the primer pairs used for PCR, their binding sites (purple) and the sequenced products (turquoise arrows). *Pac*-puromycin transacetylase gene and *hpt*-8-aza-2,6-diaminopurine hypoxanthine phosphoribosyl transferase resistance gene flanked by two Flp recombinase recognition sites (*FRT*).

Although the existence of posttranslational modifications in MCR is well established, not much is known about the enzymes introducing these modifications. It is likely that methylhistidine and methylcysteine are formed *via* S_N_2 reactions by conventional *S*-adenosyl-L-methionine (SAM) dependent *N*- and *S*-methyltransferases, respectively^20^. However, a methyl group transfer *via* an S_N_2 mechanism is not possible for the methylation of the sp^3^-hybridized electrophilic carbons C-5 of arginine and C-2 of glutamine. It was speculated that in these cases Radical SAM methyltransferases could be responsible^21^, since it is now known that these enzymes are indeed able to methylate electrophilic sp^2^- or sp^3^-carbon centres in a variety of different substrates^22,23^. Like all other Radical SAM enzymes, these methyltransferases carry at least one [4Fe-4S] cluster coordinated by the three cysteine residues of the signature motif CxxxCxxC and one SAM molecule^24^. In the course of the reaction, the iron-sulfur cluster bound SAM is reductively cleaved yielding methionine and a 5′-deoxyadenosyl radical, which initiates further radical-based chemistry and substrate transformation.

The aim of this study was to look for candidate genes potentially encoding Radical SAM methyltransferases in the genomes of methanogenic archaea and to test the hypothesis that the corresponding enzymes are responsible for the C-methylation of either arginine or glutamine.

## Results

### Identification of potential Radical SAM methyltransferase genes in the genomes of methanogens

The genes encoding the MCR subunits are invariably organised in operon structures in the genomes of methanogenic archaea. Since the production of MCR and the introduction of its posttranslational amino acid modifications must be closely linked, we started our search for potential Radical SAM methyltransferase genes by inspecting the direct neighbourhood of the *mcr* gene clusters. Strikingly, the gene annotated as “methanogenesis marker 10” (*mm10*) indeed encodes a potential Radical SAM enzyme, based on the presence of the characteristic CxxxCxxC cysteine motif, and it is located in many cases directly next to the *mcr* genes (Fig. 1c and Supplementary Table 1). As the annotation suggests, the *mm10* gene is present in almost all genomes of methanogens available so far. Exceptions of this rule are the genomes of members of the order *Methanomassiliicoccales*, the genomes of members of the proposed phylum *Bathyarchaeota*^25^, the genomes of two recently described extremely halophilic methanogens belonging to the *‘Methanonatronarchaeia’*^26^ and of Candidatus *Methanofastidiosum methylthioreducens*^27^. Moreover, whereas the *mm10* gene was found in the genome of *Methanoperedens nitroreducens* belonging to the ANME-2d subgroup of anaerobic methanotrophic archaea, it was not identified in the genome of the ANME-1 cluster archaeon ex4572_4 (Supplementary Table 1). Additionally, in 33 out of 78 genomes analysed, another suspicious gene encoding a putative cobalamin-dependent Radical SAM enzyme was identified. In some cases, this gene is located directly next to the *mm10* gene (Fig. 1c and Supplementary Table 1). Considering the fact that 5-methylarginine was present in all methanogenic MCRs experimentally characterized so far, whereas 2-methylglutamine was not always detected, we hypothesized that the *mm10* gene might encode the Radical SAM methyltransferase responsible for arginine C-methylation. Accordingly, the putative cobalamin-dependent Radical SAM enzyme might catalyse the glutamine C-methylation.

### Deletion of the gene *ma4551* encoding the putative Radical SAM methyltransferase MA4551 in *Methanosarcina acetivorans*

In order to test the hypothesis that the *mm10* gene encodes the Radical SAM methyltransferase responsible for the C-methylation of arginine, the respective gene *ma4551* from *M*. *acetivorans* was replaced by an antibiotic resistance cassette (*pac-hpt*) *via* double homologous recombination^28^. Several potential mutants were selected and the regions of the affected locus were amplified by PCR. Sequencing of the obtained PCR fragments demonstrated the replacement of *ma4551* by the *pac-hpt* cassette (Fig. 2). The *M*. *acetivorans* mutant strain lacking *ma4551* was designated Mko4551. Growth to colony size of Mko4551 demonstrated that the protein encoded by *ma4551* is not essential for the viability of *M*. *acetivorans*.

### Arg285 is not methylated in McrA from *Methanosarcina acetivorans* Mko4551

**Figure 3:**
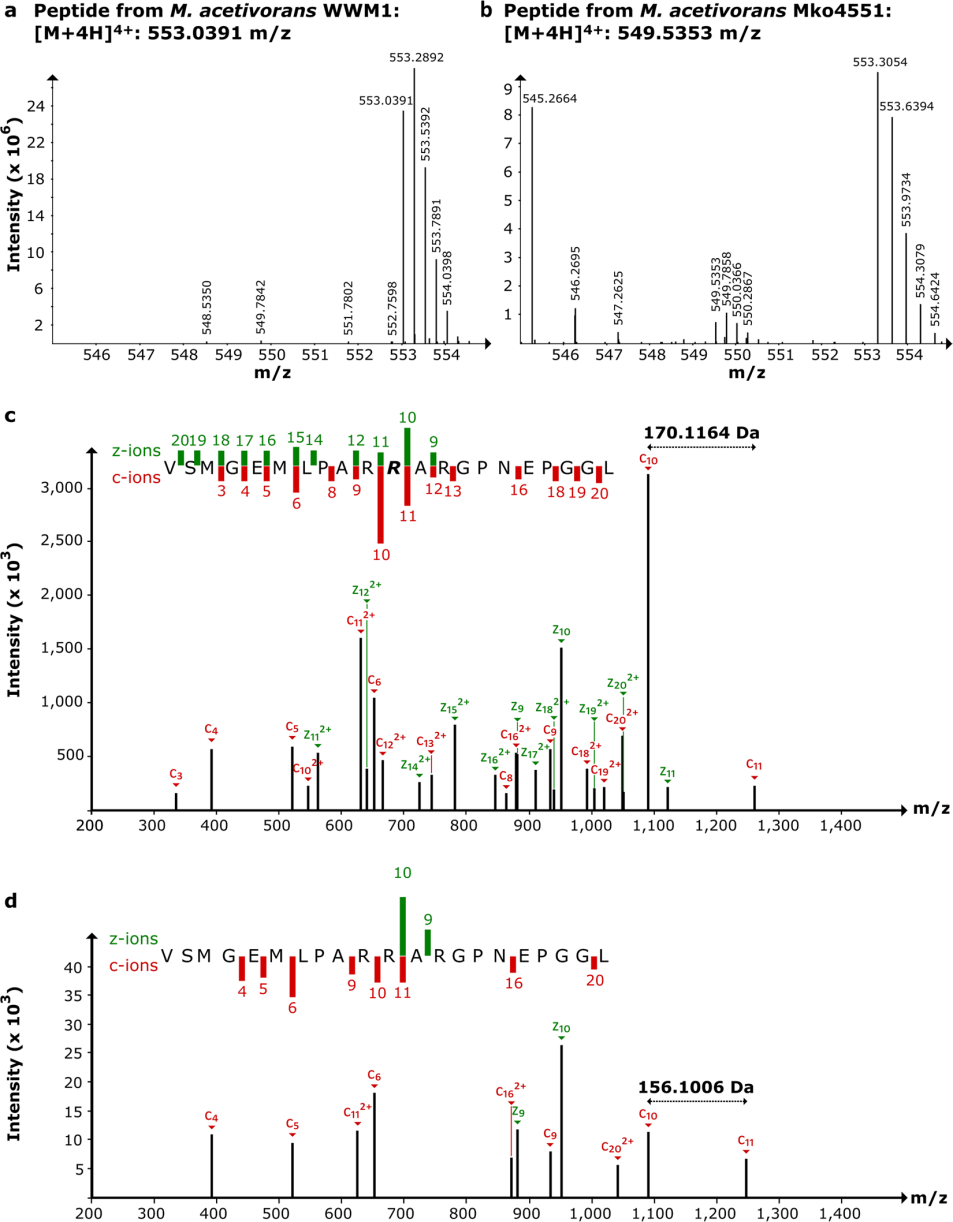
nLC/HR-ESI-MS/MS analysis of McrA from *M*. *acetivorans* WWM1 and Mko4551. (**a**) MS spectrum obtained from wild type McrA containing only the methylated precursor _275_V-**R**_285_-L_296_ ([M + 4 H]^4+^; 553.0391 m/z). (**b**) MS spectrum obtained from Mko4551 McrA containing only the non-methylated precursor _275_V-L_296_ ([M + 4 H]^4+^; 549.5353 m/z). (**c**) MS/MS spectrum of the wild type precursor, **R** = 5-methylarginine. (**d**) MS/MS spectrum of the Mko4551 precursor. R_285_ was not methylated. A complete list of calculated and measured masses for the c and z ions is given in Supplementary Table 2. The green and red bars in panels c and d indicate the presence and intensity of different MS2 fragments.

In order to obtain insights into the role of MA4551, the amino acid modifications of McrA from the *M*. *acetivorans* WWM1 (wild type) and of Mko4551 were analysed by mass spectrometry. Here, we concentrated our attention on the methylation of arginine residue 285, the potential target of MA4551. Recently, four amino acid modifications were reported to be present in McrA of *M*. *acetivorans*, namely methylhistidine (His271), methylcysteine (Cys472), thioglycine (Gly465) and didehydroaspartate (Asp470)^29^, whereas the glutamine residue 420 was unmodified as previously observed for the McrA from *Methanosarcina barkeri*^14^. In order to obtain peptide fragments from McrA containing Arg285, MCR was first enriched from cell free extracts of *M*. *acetivorans* WWM1 and of Mko4551 by anion exchange chromatography (Supplementary Figure 1). After separation of the proteins by SDS-PAGE, the bands corresponding to McrA were excised and used for in-gel digestion with chymotrypsin. The resulting peptide mixtures were then analysed by nano-liquid chromatography/high-resolution electrospray ionization tandem mass spectrometry (nLC/HR-ESI-MS/MS). In both samples, prepared either from WWM1 or from Mko4551, the peptide fragment containing Arg285 was detected by HR-ESI-MS. For the sample prepared from WWM1, the signal of this peptide displayed an m/z of 553.0391 representing the [M + 4 H]^4+^ ion of the peptide _275_VSMGEMLPAR**R**_**285**_ARGPNEPGGL_296_ with one methylation (0.06 ppm error) and corresponding to a parent peptide with a mass of 2208.1564 Da (Fig. 3a). The number of acquired charges matched to the expected number (three arginines and the N-terminus). This peptide was isolated with the quadrupole filter of the instrument and fragmented by a combination of ETD and CID fragmentation (ETciD). The resulting MS/MS spectra confirmed the methylation of Arg285 (Fig. 3c and Supplementary Table 2). In contrast, in the sample prepared from Mko4551, the _275_V-L_296_ peptide exhibited a signal at an m/z of 549.5353 for the [M + 4 H]^4+^ ion (0.28 ppm error), which corresponds to a parent peptide with a mass of 2194.1412 Da (Fig. 3b). The mass difference of 14.0158 Da between the two precursor peptides indicated the loss of a methyl group in the peptide obtained from McrA of Mko4551. Finally, the fragmentation pattern of this precursor and comparison with the MS/MS spectra of the WWM1 sample confirmed the lacking methylation of Arg285 (Fig. 3d and Supplementary Table 2).

### *M*. *acetivorans* Mko4551 shows impaired growth under stress conditions

**Figure 4:**
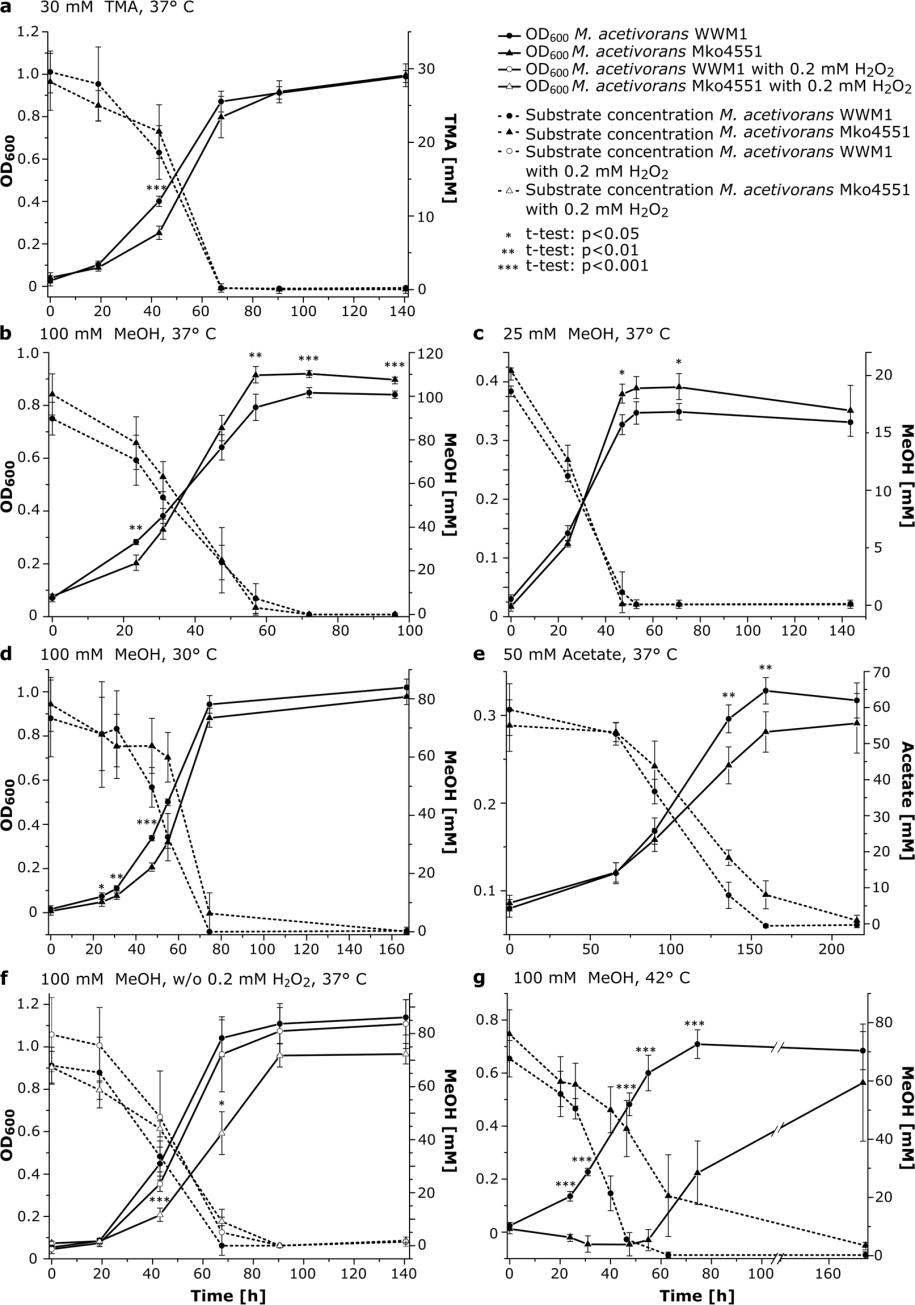
Growth behaviour and substrate consumption of *M*. *acetivorans* wt and Mko4551. Substrate type, substrate concentration and incubation temperature are presented in the panels. The growth experiments were qualitatively reproduced at least three times, shown are mean values and standard deviations of n = 4–5 independent cultures. Statistical significance was calculated *via* paired two-sample *t*-test (*p < 0.05, **p < 0.01, ***p < 0.001).

Since Arg285 is located near the CoB-binding site of MCR and is part of the substrate channel wall, the presence of arginine instead of 5-methylarginine in Mko4551 might have consequences on either the activity or stability of MCR, or both. Therefore, the growth behaviour of Mko4551 was studied under diverse conditions and compared to the growth of the wild type strain under identical conditions. *M*. *acetivorans* WWM1 and Mko4551 exhibited similar growth phenotypes for growth on methanol (MeOH; 100 mM or 25 mM) or trimethylamine (TMA; 30 mM) at 30 °C or 37 °C, respectively (Fig. 4a–d). Growth of Mko4551 in the presence of acetate was slightly affected in comparison to the wild type (Fig. 4e). However, more severe growth defects were observed for Mko4551 under stress conditions such as the presence of 0.2 mM hydrogen peroxide (H_2_O_2_) or elevated temperature (42 °C). While growth of WWM1 in the presence of H_2_O_2_ was barely affected, Mko4551 grew significantly slower under these conditions (Fig. 4f). The most drastic difference in growth between the two strains was observed during cultivation at 42 °C (100 mM MeOH). Under these conditions, Mko4551 exhibited a markedly prolonged lag phase and subsequently slower growth than WWM1 (Fig. 4g).

We also compared the MCR protein amounts in WWM1 and in Mko4551 cultivated with 100 mM MeOH at 37 °C *via* a label-free quantification method using LC-MS/MS (Supplementary Figure 2). The relative abundance of MCR was determined to be around 10% of the total cytoplasmic protein as observed previously^30^. In our study, the relative abundance of McrA in the wild type strain was 7.4 ± 1.3% in the exponential growth phase, whereas 10.6 ± 0.9% were determined for the Mko4551 strain. Thus, the amount of McrA in the Mko4551 strain was slightly elevated. However, there were no significant differences in the amounts of McrA in the two strains in the stationary growth phase. Similar observations were made for the subunits McrB and McrG. Interestingly, the amount of the heat-shock protein Hsp60-1 (MA0631) was significantly higher in Mko4551 than in the wild type strain both in the exponential and stationary growth phase.

### Methylation of Arg285 influences the thermal stability of methyl-coenzyme M reductase

**Figure 5:**
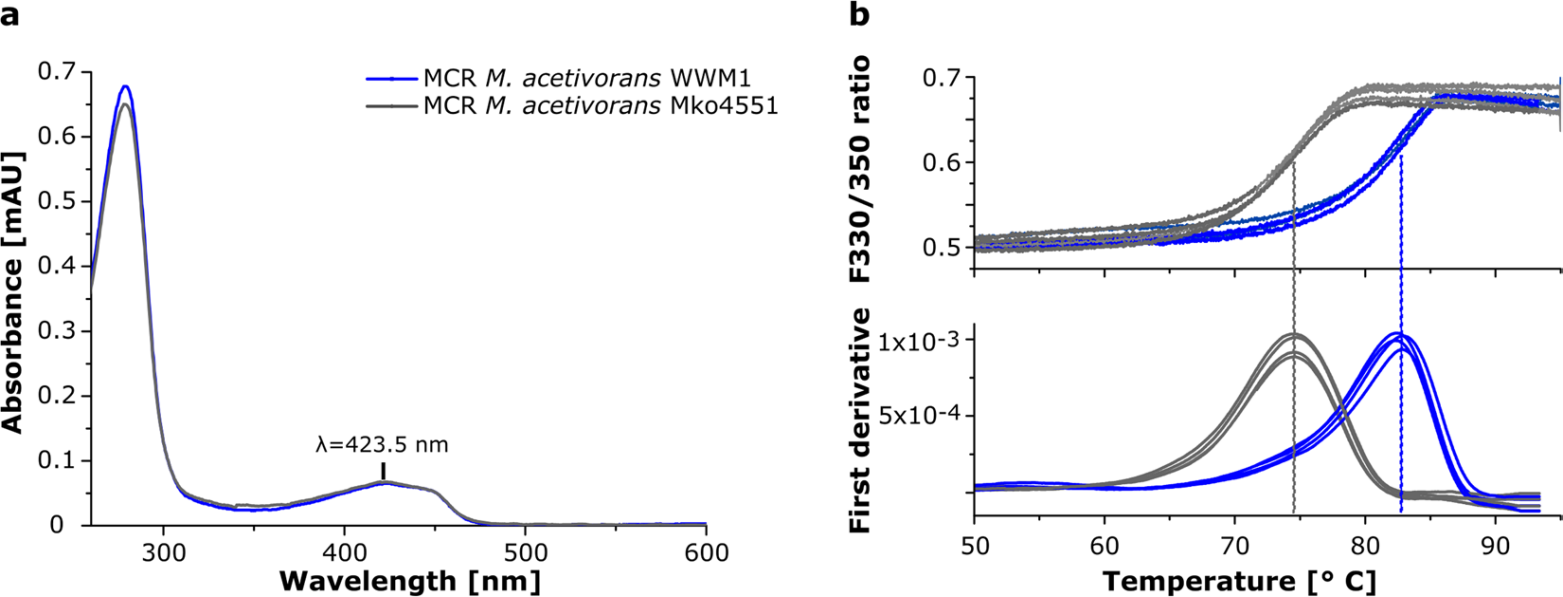
Characterization of purified methyl-coenzyme M reductase (MCR) from *M*. *acetivorans* WWM1 and Mko4551. (**a**) UV/Vis absorption spectra of purified MCR from the wild type (blue) and the Mko4551 strain (grey), both exhibiting the characteristic features of bound coenzyme F_430_ at about 423 nm. The spectra were normalised by setting the absorbance at 600 nm to zero. (**b**) T_m_ measurement of purified MCR from the wild type (blue) and the Mko4551 strain (grey) with the nanoDSF principle. Upper panel: F_330_/F_350_ ratio of the intrinsic tryptophan fluorescence plotted against the temperature, lower panel: T_m_ calculation by first derivative analysis. T_m_ (wt) = 82.6 ± 0.2 °C, T_m_ (ko) = 74.6 ± 0.1 °C. Shown are the curves of four independent measurements for each enzyme. The T_m_ values are means with standard deviation.

The observed growth phenotype of the *M*. *acetivorans* mutant at elevated temperatures and its higher Hsp60-1 contents suggested that the methylation of Arg285 might play a role for the stability and structural integrity of MCR. Therefore, enzymes purified from either WWM1 or Mko4551 were characterised for coenzyme F_430_ content and thermal stability. The UV/Vis absorption spectrum of the MCR isolated from Mko4551 was identical to that of MCR from the wild type strain, both displaying the characteristic absorption features of bound coenzyme F_430_ at 423 nm with a shoulder at about 450 nm (Fig. 5a). Moreover, the absorption ratio Abs_280_/Abs_423_ of the two isolated enzymes was almost identical suggesting that the coenzyme F_430_ content was not affected by the missing methylation of Arg285.

The thermal stability of purified MCR was assessed by recording protein melting curves using the nano differential scanning fluorimetry (nanoDSF) method, which is based on the change of the intrinsic tryptophan fluorescence during thermal denaturation of the protein. For the MCR isolated from the wild type a melting temperature (T_m_) of 82.6 ± 0.2 °C was determined. In contrast, the T_m_ of MCR from Mko4551 was at 74.6 ± 0.1 °C (Fig. 5b). Therefore, the MCR variant lacking the methylation of Arg285 is less stable during thermal denaturation than the wild type enzyme.

## Discussion

Despite the global importance of methanogenesis, several details of the process are not understood. For example, there are 17 so called “methanogenesis marker genes/proteins” (Accession: GenProp0722)^31^ that are conserved in methanogenic archaea, but are not present in non-methanogenic microorganisms. However, for many of them, the function is still not known. Here, we show that the methanogenesis marker 10 (*mm10*) is required for the introduction of the methyl modification of the 5-C-(*S*)-methylarginine in MCR. It had already been speculated in the past that the unusual C-methylations of an arginine and a glutamine residue within the McrA subunit of MCR might be introduced by Radical SAM methyltransferases^21^. Indeed, the *mm10* gene codes for a potential Radical SAM enzyme and it is present in all methanogens for which the 5-methylarginine in McrA was detected experimentally. Interestingly, the occurrence of *mm10* matches the phylogenetic analysis and classification of MCRs performed by Wagner *et al*.^17^ and Sorokin *et al*.^26^. In these studies, the MCRs from ANME-1, *Bathyarchaeota*, *Verstraetearchaeota*, *Methanomassiliicoccales*, *Candidatus ‘Methanofastidiosum methylthioreducens’* and *‘Methanonatronarchaeia’* were placed in phylogenetic clusters distinct from the MCR-clusters of other methanogens. Conspicuously, the *mm10* gene is absent in these (groups of) organisms. Therefore, we assume that the MCRs from these organisms do not carry a 5-methylarginine. Indeed, this holds true for the MCR from ANME-1 organisms^14,19^. In contrast, the genome of the ANME-2d organism *Methanoperedens nitroreducens*, closely related to the *Methanosarcinaceae*^32^, contains the *mm10* gene. Thus, the MCR from this methanotrophic archaeon most likely contains the 5-methylarginine.

It has been proposed that the methylation of arginine in McrA might be required to restrict the conformational flexibility of this residue^14,15^. This might be necessary for the formation of the inter-subunit salt bridge between the arginine and a glutamate of the neighboring McrB subunit and/or to prevent the arginine from protruding into the substrate channel^15^. Thus, the methylation might be important for the local stability and subunit interaction as well as for maintaining the ideal shape of the substrate channel for efficient substrate binding. These proposals are in line with the finding that the McrA subunit from the MCR of an ANME-1 organism lacking the methyl modification carries a 7-hydroxy-tryptophan, which compensates for the missing methyl group^19^. In our study, the missing methylation of Arg285 in MCR of Mko4551 led to impaired growth at elevated temperatures and under oxidative stress, although neither the coenzyme F_430_ content nor the cellular MCR concentration were significantly affected by the missing methyl group. However, the determination of the thermal stability of MCR demonstrated that the enzyme lacking the methylation of Arg285 is less stable than the wild type enzyme (T_m_ difference of 8 °C). Thus, despite being not essential *in vivo*, the 5-methylarginine plays a role for the stability and structural integrity of MCR. Such a role is supported by the observed elevated amounts of the chaperonin component Hsp60-1 in Mko4551 compared to the wild type. However, an additional role of the 5-methylarginine for efficient substrate binding and in fine-tuning the enzyme activity cannot be ruled out at present. In the future, determination of the structure of the MCR variant in combination with its enzymatic characterisation will provide further insights into the precise role of the unusual methylated arginine.

The presence of an unmodified Arg285 in the McrA subunit of the *M*. *acetivorans* Mko4551 strain shows that the predicted Radical SAM enzyme MA4551 is involved in the posttranslational methylation of this residue, most likely by acting as the responsible methyltransferase. So far, Radical SAM methyltransferases have been grouped into four classes according to their domain architecture, cofactor requirement and postulated enzyme mechanism^22,33,34^. Class A enzymes represented by RlmN and Cfr use two highly conserved cysteine residues to accomplish the methylation of sp^2^-hybridized carbon centres of adenosine moieties in several RNAs^35–37^. Class B Radical SAM methyltransferases constitute the largest and most versatile class including enzymes that methylate either sp^2^- or sp^3^-hybridized carbons or phosphinate phosphorous atoms. These enzymes contain an N-terminal cobalamin binding domain and rely on methylcobalamin as the direct methyl group donor^38–40^. Class C Radical SAM methyltransferases catalyse the methylation of sp^2^-hybridized carbons and are characterized by a C-terminal HemN-like domain^22,33^. Thus, the simultaneous binding of two SAM molecules is proposed for Class C enzymes^41–43^. Further, it is suggested that the SAM molecule bound to the [4Fe-4S] cluster gives rise to the 5′-deoxyadenosyl radical, whereas the second SAM molecule may be the source of the methyl group^44,45^. Finally, the recently described Class D enzyme MJ0619 from *Methanocaldococcus jannaschii* contains two CxxxCxxC motifs and is proposed to catalyse two methylation reactions of sp^3^-hybridized carbons during the biosynthesis of methanopterin. In this case, 5,10-methylenetetrahydromethanopterin is suggested to serve as the source of the methyl groups rather than SAM^34^. The putative Radical SAM methyltransferase MA4551 does not share significant amino acid sequence similarity with any of the so far described Radical SAM methyltransferases. MA4551 does also not exhibit the two strictly conserved cysteine residues of Class A members, it lacks the N-terminal cobalamin binding domain characteristic for Class B methylases and it does not share any sequence similarities with HemN or Class C methylases or the Class D enzymes. Accordingly, a phylogenetic tree derived from an alignment of multiple amino acid sequences shows that MA4551 belongs to a new and uncharacterized Radical SAM methyltransferase group rather than to the described classes (Supplementary Figure 3). Besides the known Radical SAM motif CxxxCxxC, MA4551 and all other “methanogenesis marker 10” enzymes contain a second strictly conserved cysteine motif, CxxCx_5-6_CxxC (Supplementary Figure 4). Whether the cysteines of this motif serve as the ligands for a second iron-sulfur cluster or another purpose, remains to be established in future experiments.

## Methods

### General

All chemicals, media ingredients and other reagents were purchased from Sigma-Aldrich and Carl Roth GmbH & Co. KG. All chemicals used for mass spectrometry were in LC-MS grade. Restriction enzymes and DNA polymerase were obtained from New England BioLabs GmbH. Bacterial and archaeal strains, plasmids and oligonucleotide primers (Seqlab-Sequence Laboratories Göttingen GmbH) used in this study are listed in Supplementary Tables 3 and 4.

### Bioinformatical analysis

For the analysis and comparison of methanogen genomes, the Microbial Genome Database for Comparative Analysis (MBGD) was used^46,47^. For genome clustering, 60 completely sequenced genomes of methanogenic archaea were used with the default parameters of the database. Homologous genes were identified with the “orthologous cluster” tool. In total, 64 genomes of methanogens and 1 genome of an ANME-2d archaeon were analysed using the MBGD. Additionally, the genomic data of some methanogens not present in the MBGD, of several ANME archaea, of methanogens belonging to the archaeal phyla *Bathyarchaeota* and *Verstraetearchaeota*, and of two extremely halophilic methanogens were searched for the presence of the *mm10* gene and the gene for the putative cobalamin-dependent Radical SAM methyltransferase by using the blastp tool of the National Centre for Biotechnology Information (NCBI)^48^ or by using the Artemis software^49^.

The evolutionary relationship between different Radical SAM methyltransferases was inferred by using the Neighbour-Joining method^50^. The analysis involved 25 amino acid sequences derived from the NCBI database^48^. All positions containing gaps and missing data were eliminated resulting in a total of 262 positions in the final dataset. The MEGA7 software was used to conduct the multiple amino acid sequence alignment using the ClustalW algorithm and to generate an evolutionary tree^51^. The evolutionary distances were computed using the Poisson correction method and are in the units of the number of amino acid substitutions per site^52^.

### Strains and cultivation

*Escherichia coli* strains were grown under standard conditions in LB broth at 37 °C with 100 µg mL^−1^ ampicillin. *M*. *acetivorans* strains were grown (in single-cell morphology) in high-salt (HS) liquid medium as previously described, supplemented with either 100 mM or 25 mM methanol (MeOH), 30 mM trimethylamine (TMA) or 50 mM sodium acetate (Ac) under strict anaerobic conditions^53^. Cultures were grown in sealed bottles with either N_2_ (0.4 bar) in the headspace for growth with MeOH or an N_2_/CO_2_ mixture (0.4 bar) for growth with Ac as described in previous studies^54,55^. Cultivation on solid medium containing 1.5% (w/v) Bacto agar was conducted as previously described^56^. Plating of *M*. *acetivorans* on solid media was carried out in an anaerobic glove box (Coy Laboratory Products) in an N_2_:CO_2_:H_2_ atmosphere (78:18:4% [v/v]). Agar plates were incubated in pressurized anaerobic jars at 37 °C as previously described^57^. 2 µg mL^−1^ puromycin from an anaerobic and sterile stock solution was added to select for the puromycin transacetylase (*pac*) gene. Growth of *M*. *acetivorans* was monitored by measuring the optical density at 600 nm (OD_600_).

### Quantification of methanol

MeOH concentrations in culture media were determined by gas chromatography (GC) using an Agilent Technologies 7820 GC system linked to a PAL LHT-xt autosampler with a split ratio of 5:1. The samples were separated with a Zebron ZB-1 (length 60 m; inner diameter 0.32 mm; film thickness 1 µm) column (Phenomenex Ltd.). 500 µL of each sample were transferred into GC vials and crimped. Then the samples were heated for 10 min at 80 °C prior to injection of 2 mL gas phase. The injector temperature was set to 220 °C. For the separation of compounds, the oven temperature was set and hold for 5 min at 40 °C and elevated at a rate of 30 °C min^−1^ to 220 °C. The FID detector flow was set to 1.5 mL min^−1^ and the FID makeup flow to 20 mL min^−1^. For the standard curve, MeOH solutions with known concentrations between 0 and 50 mM were used.

### Quantification of acetate

For the quantification of acetate in culture media, a colorimetric assay kit from abcam® was used according to the manufacturer’s instructions. Culture samples were centrifuged for 5 min at 13,000 rpm (Eppendorf centrifuge 5424 R) and 4 °C and diluted 100-fold prior to use. Acetate solutions between 0 and 0.5 mM were prepared for the standard curve. 25 µL of each culture sample or standard solution were mixed with 25 µL of the reaction mix and incubated at RT for 40 min. Finally, the absorbance of the solutions was measured at 450 nm in a 96 well plate using the iMark Microplate Absorbance Reader (BioRad).

### Quantification of trimethylamine

TMA concentrations in culture media were determined by GC using an Agilent Technologies G1530A FID system linked to a COMBI PAL autosampler (CTC Analytics AG) with a split ratio of 5:1. The samples were separated with an HP-5 column (length 30 m; inner diameter 0.32 mm; film thickness 0.25 µm, Agilent Technologies). 500 µL of each sample were transferred into GC vials and crimped. The samples were heated for 10 min at 80 °C prior to injection of 2 mL gas phase. The injector temperature was set to 220 °C. The oven temperature was set and hold for 5 min at 40 °C, elevated at a rate of 35 °C min^−1^ to 140 °C, then at 10 °C min^−1^ to 190 °C and finally to 220 °C within 1 min. The FID detector flow was set to 0.5 mL min^−1^ and the FID makeup flow amounted to 20 mL min^−1^. For the standard curve, TMA solutions with known concentrations between 0 and 50 mM were used.

### Construction of *ma4551* knockout plasmids

Approximately 1 kb up- and downstream regions flanking the gene *ma4551* were PCR-amplified using the Q5® High-Fidelity DNA polymerase and oligonucleotide primers containing 15 nucleotide overhangs listed in Supplementary Table 3. The obtained PCR fragments were cloned into pJK301^58^ using the In-Fusion® HD Cloning Kit (Takara Bio USA, Inc.) according to the manufacturer’s instructions. The upstream region of *ma4551* was PCR-amplified using the primer pair UP-fw und UP-rev and subsequently cloned into the plasmid pJK301, digested with *HindIII* and *XhoI* restriction enzymes, yielding the plasmid pJK_UP. The downstream region of *ma4551* was PCR-amplified with the primers DOWN-fw and DOWN-rev and cloned into the pJK_UP vector digested with *BamHI* and *SpeI*. The resulting plasmid pJK301_ma4551 was linearized using *ApaI* and *AleI*. The linear fragment containing the up- and downstream regions of *ma4551*, the *pac-hpt* cassette and the FRT sites was purified using the GeneJET PCR Purification Kit (Thermo Fisher Scientific) and transferred into *M*. *acetivorans* WWM1 for double homologous recombination.

### Transformation methods

*E*. *coli* DH10B was transformed by electroporation at 1800 V using an Eppendorf Eporator®. The transformation of *M*. *acetivorans* WWM1 was carried out by a polyethylene glycol-mediated approach^59^. About 2 µg of the linearized plasmid pJK301_ma4551 was used for the transformation procedure.

### Validation of the *M*. *acetivorans* mutant strain

The genotype of the *M*. *acetivorans* mutant strain Mko4551 was verified by PCR using the primer pairs Ma5/Ma6 and DD15/DD73 (Supplementary Table 3 and Fig. 2). The respective PCR products were analysed by agarose gel electrophoresis, purified using the GeneJET PCR Purification Kit (Thermo Fisher Scientific) and sequenced (SeqLab, Microsynth AG).

### Purification of methyl-coenzyme M reductase

For the purification of native MCR from *M*. *acetivorans* WWM1 and from Mko4551, the cells were harvested by centrifugation for 10 min at 10,000 rpm (Eppendorf centrifuge 5804 R) and 10 °C. Cell harvesting and all purification steps were conducted in an anaerobic chamber (Coy Laboratories) using degassed, anaerobic buffer. The cells were lysed in 50 mM MOPS, pH 7.0 using 0.1 mm zirconia beads with FastPrep-24™ (6 m s^−1^, 30 s). MCR was enriched by anion exchange chromatography using a MonoQ 5/50 GL column (GE Healthcare Life Sciences). The soluble protein fraction was applied to the column equilibrated with 50 mM MOPS, pH 7.0. After washing with 50 mM MOPS, pH 7.0, the bound proteins were eluted using a linear NaCl gradient (0 to 1 M NaCl within 30 ml) at a flow rate of 1 mL min^−1^. Elution of the proteins was followed by monitoring the absorbance at 280 and 430 nm. The MCR complex eluted at 0.4 M NaCl as determined by its absorbance at 430 nm. The fractions containing the MCR complex were pooled and concentrated with Amicon® Ultra-4 centrifugal filter units (MWCO 100 kDa). MCR was further purified *via* gel filtration chromatography using a Superdex 200 10/300 GL column (GE Healthcare Life Science) equilibrated with 50 mM MOPS, pH 7.0. Fractions exhibiting maximal absorbance at 430 nm were collected and the purity and integrity of the MCR protein complex was assessed by SDS-PAGE (Supplementary Figure 1).

### UV/Vis absorption spectroscopy

The UV/Vis absorption spectra of MCR (4 µM) from *M*. *acetivorans* WWM1 and from Mko4551 were recorded under anaerobic conditions using a Jasco V-650 spectrophotometer (Pfungstadt, Germany). The scanning range was 200–800 nm and 50 mM MOPS, pH 7.0 was used as a blank.

### Analysis of methyl-coenzyme M reductase stability

The thermal stability of MCR was assessed by differential scanning fluorimetry (nanoDSF). NanoDSF is a label-free method based on the intrinsic tryptophan and tyrosine fluorescence of proteins. 10 µL of purified MCR (16 µM) either from WWM1 or from Mko4551 were anaerobically loaded into the capillaries (Monolith NT.LabelFree Zero Background Standard Treated Capillaries) and sealed with wax. The measurements were performed using a Prometheus NT.48 (NanoTemper Technologies) at an excitation power of 15%. The fluorescence emission intensities at 330 nm and 350 nm (excitation at 280 nm) between 20–95 °C (ramp 1 °C min^−1^) were measured. Melting curves were generated by plotting the fluorescence ratios of F_330_/F_350_ against the temperature. The melting temperature T_m_ that describes the thermal unfolding transition midpoint was determined by first derivate analysis.

### Sample preparation for mass spectrometry

For mass spectrometric analysis, the MCR enriched fractions after anion exchange chromatography were used. The MCR containing protein solution was developed on 10% (w/v) SDS polyacrylamide gels. The gels were stained with Coomassie Brilliant Blue R-250. The protein bands at the height of 60 kDa corresponding to McrA were excised. The proteins within the gel slices were destained with acetonitrile, reduced with 10 mM dithiothreitol, alkylated with 100 mM iodoacetamide and finally digested with 0.1 µg chymotrypsin (Promega) at 25 °C for 18 h. The resulting peptides were extracted with 50% (v/v) acetonitrile (ACN) and 5% (v/v) formic acid and dried. The peptides were resuspended in 10 µL of 0.1% formic acid and desalted with C18 ZipTip® Pipette Tips (Merck Millipore). Finally, the samples were dried in a vacuum centrifuge and dissolved in 20 µL of 0.1% formic acid.

### Mass spectrometry

5 µL of each sample were injected into a nano-UPLC system (UltiMate 3000 RSLCnano System, Thermo Fisher Scientific) equipped with an Acclaim PepMap® 100 75 µm × 25 cm C18 column and resolved with an Orbitrap Fusion™ mass spectrometer (Thermo Fisher Scientific). The peptides were separated at a flow rate of 0.3 µL min^−1^ by applying a 60 min gradient from 3.2% to 44% ACN in water containing 0.1% formic acid. The mass spectrometer was run in positive-ionization mode with spray voltage set at 2.2 kV and source temperature at 220 °C. For full MS1 scans the instrument operated over a mass range of m/z 300–2000 with detection in the Orbitrap at a resolution of 240,000. The most intense ions above a threshold ion count of 5.0E4 were selected for fragmentation with the quadrupole using an isolation window of 1.6 m/z. Fragmentation was achieved by ETciD (ETD reaction time 100 ms, CID collision energy 35%). Fragment ion spectra were acquired in the Orbitrap at a resolution of 60,000 and a maximum injection time of 120 ms for Orbitrap MS2 detection.

### Protein and peptide identification

The raw mass spectrometric data were converted to mgf-files using MSConvert. SearchGUI software (v3.2.17) and the OMSSA search engine were used for peptide identification. The data were searched against the *M*. *acetivorans* proteome database obtained from UniProt (Taxon identifier 188937). Through ETciD fragmentation c and z ions were obtained. A precursor ion mass tolerance of 10 ppm was used at the MS1 level and up to two missed cleavages were allowed. The fragment ion mass tolerance was set to 0.2 Da for the Orbitrap MS2 detection method. The oxidation of methionine and the methylation of Arg, His and Gln were defined as variable modifications. Carbamidomethylation on cysteines was set as a fixed modification. The false discovery rate (FDR) in peptide identification was limited to a maximum of 0.01 by using a decoy database. The analysed data were visualised with the PeptideShaker software (v1.16.9) (CompOmics, Ghent University).

### Label-free protein quantification

For quantification, the precursor ion areas in a node-based processing and consensus workflow in Proteome Discoverer 2.2.0.338 (Thermo) were used. For this, cells of *M*. *acetivorans* WWM1 and Mko4551 grown with 100 mM MeOH at 37 °C were taken from the exponential and stationary growth phases, harvested *via* centrifugation and lysed as described above. The protein content of the cell free extracts was determined by the Bradford method^60^. 25 µg of each protein solution were pre-cleaned *via* application on a 10% (w/v) SDS-polyacrylamide gel. After a short electrophoresis during which the protein samples completely migrated into the stacking gel for a few millimeters, the complete protein smear was cut out. After that, the proteins were in-gel digested with trypsin and analysed in triplicate with nLC/HR-ESI-MS/MS as described above injecting 3 µl of each sample. For quantification, each of the data sets was run separately using a standard processing and a basic consensus workflows delivered by Thermo Fisher Scientific. The SequestHT search was performed against the entire *M*. *acetivorans* protein database (NCBI accession number NC_003552). Peptide search was conducted allowing for up to two missed trypsin cleavages. Carbamidomethylation (+57.021 Da) of cysteine residues was set as fixed modification, and oxidation of methionine residues (+15.9949 Da), methylation of His, Gln, Arg (+14.016) were set as variable modifications. The precursor mass tolerance was set to 3 ppm and product ions were searched at 0.2 Da tolerances. Peptide spectral matches (PSM) were validated using the Percolator algorithm, based on q-values of 1% FDR. The areas of the ions in the MS1 scan were calculated using the Minora Feature Detector node. Further quantification was performed using the consensus workflow performing retention time alignment and feature mapping. The Feature Mapper node groups detected features from the processing workflow allowing quantification across all raw files thereby performing gap filling. In ‘Peptide and Protein Filter’ node the minimum number of peptide sequences was set to two.

### Data availability

Almost all data generated and analysed during this study are included in this published article (and its Supplementary Information files). Additional mass spectrometric data generated during the current study are available from the corresponding author on reasonable request.

## Electronic supplementary material

Supplementary Material
