# Supplementary material for: Identification of a unique Radical SAM methyltransferase required for the sp3-C-methylation of an arginine residue of methyl-coenzyme M reductase
Source: Sci Rep. 2018 May 9;8:7404. doi: 10.1038/s41598-018-25716-x (PMC5943407; doi:10.1038/s41598-018-25716-x)
Supplement: Supplementary file 1 — Supplementary Material [file 41598_2018_25716_MOESM1_ESM.pdf]

Supplementary Information of the manuscript:

Identification of a unique Radical SAM methyltransferase required for the sp3-C-methylation of an arginine residue of methyl-coenzyme M reductase

Darja Deobald[1], Lorenz Adrian[2,3], Christian Schöne[4], Michael Rother[4], Gunhild Layer[1*]

[1]Leipzig University, Institute of Biochemistry, Brüderstraße 34, 04103 Leipzig, Germany

[2]Helmholtz Centre for Environmental Research – UFZ, Isotope Biogeochemistry, Permoserstraße 15, 04318 Leipzig, Germany

[3]Technische Universität Berlin, Chair of Geobiotechnology, Ackerstraße 74, 13355 Berlin, Germany

[4]Technische Universität Dresden, Institute of Microbiology, Zellescher Weg 20b, 01217 Dresden, Germany

Running title: Arginine methylation of methyl-coenzyme M reductase

*Corresponding author:

Gunhild Layer, Leipzig University, Institute of Biochemistry, Brüderstraße 34, 04103 Leipzig, Germany, Tel.: +49 (0) 341 97 36996, Fax: +49 (0) 341 97 36919, E-Mail: gunhild.layer@uni-leipzig.de

Keywords: Radical SAM methyltransferase, sp^3-C-methylation, methanogenesis, methyl-coenzyme M reductase, *Methanosarcina acetivorans*, methanogenesis marker protein 10, MCR, McrA, 5-C-(*S*)-methylarginine.

Supplementary Table 1: List of organisms analyzed for the presence of the *mm10* gene and the gene for the potential B12-dependent Radical SAM methyltransferase (B12 RS MT). The locus-tags for the genes encoding the MCR subunits are also listed.

	Organism / genome	Locus_tag prefix	*mm10* gene[a]	*mcrA*	*mcrB*	*mcrG*	B12 RS MT	Methyl-Arg[b]	Methyl-Gln[b]
1	*Methanobacterium formicicum* BRM9	BRM9	**_RS04725**	_RS04700<br>_RS10890	_RS04720<br>_RS10875	_RS04705<br>_RS10885	_RS04730		
2	*Methanobacterium sp. AL-21*	METBO	**_0348**	_0353<br>_2281	_0349<br>_2278	_0352<br>_2280	—		
3	*Methanobacterium sp. SWAN-1*	MSWAN	**_2061**	_2056	_2060	_2057	—		
4	*Methanobacterium sp. MB1*	MBMB1	**_RS08205**	_RS08180<br>_RS08890	_RS08200<br>_RS08875	_RS08185<br>_RS08885	_RS08210		
5	*Methanobrevibacter ruminantium* M1	MRU	**_1929**	_1924	_1928	_1925	—		
6	*Methanobrevibacter smithii* DSMZ 861	MSM	**_1020**	_1015<br>_0902	_1019<br>_0905	_1016<br>_0903	—		
7	*Methanobrevibacter sp. AbM4*	ABM4	**_1531**	_1526	_1530	_1527	—		
8	*Methanosphaera stadtmanae* DSM 3091	MSP	_0298	_0321	_0318	_0320	—		
9	*Methanothermobacter marburgensis* Marburg	MTBMA	**_C15530**	_C15480<br>_C15120	_C15520<br>_C15150	_C15490<br>_C15130	_C15540	yes<br>yes	yes<br>yes
10	*Methanothermobacter sp. CaT2*	MTCT	**_RS05395**	_RS05370<br>_RS05195	_RS05390<br>_RS05210	_RS05375<br>_RS05200	_RS05400		
11	*Methanothermobacter thermautotrophicus* Delta H	MTH	**1170**	1164<br>1129	1168<br>1132	1165<br>1130	1171		
12	*Methanothermobacter wolfeii*	MWIV6	**_RS07565**	_RS07540	_RS07560	_RS07545	_RS07570	yes	yes
13	*Methanothermus fervidus* DSM 2088	MFER	**_0789**	_0784<br>_0734	_0788<br>_0731	_0785<br>_0733	_0790		
14	*Methanocaldococcus fervens* AG86	MEFER	**_0933**	_0938	_0934	_0937	_1125		
15	*Methanocaldococcus infernus* ME	METIN	_0607	_0281	_0285	_0282	_0104		
16	*Methanococcus jannaschii* DSM 2661	MJ	**_0841**	_0846<br>_0083	_0842<br>_0081	_0845<br>_0082	_0865	yes	yes
17	*Methanocaldococcus sp. FS406-22*	MFS40622	**_1320**	_1315<br>_0424	_1319<br>_0426	_1316<br>_0425	_1297		
18	*Methanocaldococcus sp. JH146*	JH146	**_RS05395**	_RS05370<br>_RS02560	_RS05390<br>_RS02570	_RS05375<br>_RS02565	_RS05265		
19	*Methanocaldococcus vulcanius* M7	METVU	**_0144**	_0149<br>_1194	_0145<br>_1192	_0148<br>_1193	_0282		
20	*Methanotorris igneus* Kol 5	METIG	**_1232**	_1237<br>_0445	_1233<br>_0442	_1236<br>_0444	_0268		
21	*Methanotorris formicicus* DSM 16983	MetfoDRAFT	**_0543**	_0538<br>_1092	_0542<br>_1089	_0539<br>_1091	—	yes<br>yes	yes<br>yes
22	*Methanococcus aeolicus* Nankai-3	MAEO	**_1262**	_1268	_1264	_1267	_0800		
23	*Methanococcus maripaludis* S2	MMP	**1554**	1559	1555	1558	1350		
24	*Methanococcus vannielii* SB	MEVAN	**_0867**	_0872	_0868	_0871	_0661		
25	*Methanococcus voltae* A3	MVOL	**_1123**	_1128	_1124	_1127	_0045<br>_0105	n.f.	yes
26	*Methanothermococcus okinawensis* IH1	METOK	**_0961**	_0956	_0960	_0957	_0502		

27	*Methanothermococcus thermolithotrophicus* DSM 2095	F555	_RS0101870	_RS0108305	_RS0108290	_RS0108300	_RS0101405	yes	yes
28	*uncultured methanogenic archaeon RC-I*	LRC180	LRC180	RCIX2063	RCIX2059	RCIX2062	—		
29	*Methanocella conradii*<br>HZ254	MTC	_1160	_0908	_0904	_0907	—		
30<br>-	*Methanocella paludicola*<br>SANAE	MCP	_1925	_0516	_0512	_0515	—		
31	*Methanocorpusculum labreanum*<br>Z	MLAB	_1316	_1068<br>_1561	_1066<br>_1565	_1067<br>_1562	—		
32	*Methanoculleus bourgensis*<br>MS2	BN140	_2007	_1521<br>_1738	_1524<br>_1734	_1522<br>_1737	_0208		
33	*Methanoculleus marisnigri*<br>JR1	MEMAR	_1632	_0378<br>_0613	_0375<br>_0617	_0377<br>_0614	_0391		
34	*Methanoculleus thermophilus*<br>DSM 3915	MCUTH	_RS00145	_RS07565<br>_RS06280	_RS07580<br>_RS06260	_RS07570<br>_RS06275	_RS07515	yes	yes
35	*Methanofollis liminatans*<br>DSM 4140	METLI	_1634	_0498<br>_1710	_0502<br>_1713	_0499<br>_1711	_2127		
36	*Methanoplanus petrolearius*<br>DSM 11571	MPET	_2296	_2415<br>_1991	_2419<br>_1993	_2416<br>_1992	_2151		
37	*Methanomicrobium mobile*<br>BP	T502	_RS08355	_RS04680	_RS04700	_RS04685	—		
38	*Methanoplanus limicola*<br>DSM 2279	METLIM	_2412	_2882	_2886	_2883	_2560		
39	*Methanolinea tarda*<br>NOBI-1	METTADRAFT	_RS00730	_RS08625	_RS08605	_RS08620	—		
40	*Methanoregula boonei*<br>6A8	MBOO	_1781	_0582	_0586	_0583	—		
41	*Methanoregula formicicum*<br>SMSP	METFOR	_1315	_0874<br>_2609	_0871<br>_2605	_0873<br>_2608	—		
42	*Methanosphaerula palustris*<br>E1-9c	MPAL	_1909	_2309	_2305	_2308	—		
43	*Methanospirillum hungatei*<br>JF-1	MHUN	_2512	_2148	_2144	_2147	_2167		
44	*Methanoperedens nitroreducens*<br>(ANME-2d)	ANME2D	_RS07350	_RS05350	_RS05335	_RS05345	—		
45	*Methanosaeta concilii*<br>GP6	MCON	_1242<br>_2443	_0759	_0762	_0760	_0758		
46	*Methanosaeta harundinacea*<br>6Ac	MHAR	**_0503**	_0498	_0495	_0497	_0502		
47	*Methanosaeta thermophila*<br>PT	MTHE	_0508	_0569	_0572	_0570	_0558		
48	*Methanococcoides burtonii*<br>DSM 6242	MBUR	**_2422**	_2417	_2421	_2418	—		
49	*Methanococcoides methylutens*<br>MM1	MCMEM	**_RS03025**	_RS03000	_RS03020	_RS03005	—		
50	*Methanohalobium evestigatum*<br>Z-7303	METEV	**_0908**	_0902	_0906	_0903	—		
51	*Methanohalophilus mahii*<br>DSM 5219	MMAH	**_0617**	_0612	_0616	_0613	—		
52	*Methanolobus psychrophilus*<br>R15	MPSY	**_0834**	_0828	_0832	_0829	—		
53	*Methanomethylovorans hollandica*<br>DSM 15978	METHO	**_1665**	_1670	_1666	_1669	—		
54	*Methanosalsum zhilinae*<br>DSM 4017	MZHIL	_1044	_0857	_0853	_0856	—		
55	*Methanosarcina acetivorans*<br>C2A	MA	**4551**	4546	4550	4547	—	yes	no
55	*Methanosarcina barkeri*	MBAR	**_A0898**	_A0893	_A0897	_A0894	—	yes	no

	Fusaro								
56	*Methanosarcina horonobensis* HB-1	MSHOH	**_RS00435**	_RS00460	_RS00440	_RS00455	—		
57	*Methanosarcina lacustris* Z-7289	MSLAZ	**_RS00875**	_RS00850	_RS00870	_RS00855	—		
58	*Methanosarcina mazei* Goe1	MM	**_1245**	_1240	_1244	_1241	—		
59	*Methanosarcina siciliae* C2J	MSSAC	**_RS22375**	_RS22400	_RS22380	_RS22395	—		
60	*Methanosarcina sp. Kolksee*	MSKOL	**_RS17995**	_RS17970	_RS17990	_RS17975	—		
61	*Methanosarcina sp. WH1*	MSWH1	**_RS00455**	_RS00480	_RS00460	_RS00475	—		
62	*Methanosarcina thermophila* CHTI-55	MSTHC	**_1650**	_1645	_1649	_1646	—		
63	*Methanosarcina vacuolata* Z-761	MSVAZ	**_RS18095**	_RS18070	_RS18090	_RS18075	—		
64	*Methermicoccus shengliensis* DSM 18856	BP07	_RS05325	_RS04365	_RS04345	_RS04360	—		
65	*Methanopyrus kandleri* AV19	MK	**0650**	0655	0651	0654	0797	yes	yes
66	*Methanomethylophilus alvus* Mx1201	MMALV	—	_04000	_03970	_03990	—		
67	*Methanoplasma termitum* MpT1	MPT1	—	_RS01010	_RS00995	_RS01005	—		
68	*Methanomassiliicoccus intestinalis* Issoire-Mx1	H729	—	_07305	_07320	_07310	—		
69	*Methanonatronarchaeum thermophilum* AMET1	AMET1	—	_1463	_1459	_1461	—		
70	C. *'Methanohalarchaeum thermophilum'* HMET1	BTN85	—	_0917	_0914	_0916	—		
71	C. *'Methanofastidiosum methylthioreducens'* U1lsi0528_Bin055	AMQ22	—	_00302	_00305	_00303	—		
72	C. *'Methanomethylicus mesodigestum'*	V1_s30	—	_89	_91	_90	—		
73	C. *'Methanomethylicus oleusabulum'*	V3_s66	—	_30	_28	_29	—		
74	C. *'Methanosuratus petracarbonis'*	V4_s73	—	_10	_12/13	_11	—		
75	C. Bathyarchaeota archaeon BA1	AOA65	—	_0408	_0407	_0406	—		
76	C. Bathyarchaeota archaeon BA2	AOA66	—	_1761	_1769	_1770	_1763		
77	ANME-1 cluster archaeon ex4572_4	B6V00	—	_01170	_01180	_01175	—	no	no
78	ANME-2 cluster C. '*Syntrophoarchaeum butanivorans'*	SBU	—	_000718 _001343	_000719 _001341	_001009 _001327	—		

[a]*mm10* genes in direct vicinity to the *mcr* operon are highlightled in bold.

[b]experimental evidence for the presence (yes) or absence (no) of 5-methylarginine and 2-methylglutamine.

Supplementary Table 2: nLC/HR-ESI-MS/MS data for the precursor peptide $_{275}$V-L$_{296}$ of McrA from *M. acetivorans* WWM1 and Mko4551. The presence of the Arg285 methylation in the wild-type (wt) strain and the absence of the modification in the Mko4551 strain (ko) were included for the calculation of the [M+H]$^+$. The determined fragment masses (Da) of the respective precursor peptides are shown. The amino acid (AA) residue Arg285 is underlined, c ions are highlighted in green and z ions in red.

**$_{275}$V-R$_{285}$-L$_{296}$ wt: expected**		**wt: measured [M+H]$^+$**		**c**	**AA**	**z**	**$_{275}$V-L$_{296}$ ko: expected [M+H]$^+$**		**ko: measured [M+H]$^+$**	
117.1023				**1**	**V**	**21**	117.1023			
204.1342	2094.0434			**2**	**S**	**20**	204.1343	2080.0234		
335.1748	2007.0114	**335.1748**		**3**	**M**	**19**	335.1748	1992.9914		
392.1963	1875.9709	**392.1962**		**4**	**G**	**18**	392.1963	1861.9509	**392.1962**	
521.2389	1818.9494	**521.2388**		**5**	**E**	**17**	521.2389	1804.9294	**521.2388**	
652.2793	1688.9068	**652.2793**		**6**	**M**	**16**	652.2793	1675.8868	**652.2793**	
765.3634	1558.8663			**7**	**L**	**15**	765.3634	1544.8463		
862.4162	1445.7823	**862.4161**		**8**	**P**	**14**	862.4162	1431.7623		
933.4533	1348.7295	**933.4532**	**1348.7318**	**9**	**A**	**13**	933.4533	1334.7095	**933.4532**	**1334.7162**
1089.5544	1277.6924	**1089.5533**	**1277.6947**	**10**	**R**	**12**	1089.5544	1263.6724	**1089.5520**	**1263.6791**
1259.6755	1121.5913	**1259.6697**	**1121.5936**	**11**	**R**	**11**	1245.6555	1107.5713	**1245.6526**	**1107.5780**
1330.7126	951.4702		**951.4768**	**12**	**A**	**10**	1316.6926	951.4702		**951.4768**
1486.8137	880.4331		**880.4397**	**13**	**R**	**9**	1472.7937	880.4331		**880.4397**
1543.8352	724.3319		**724.3386**	**14**	**G**	**8**	1529.8152	724.3319		
1640.8880	667.3105			**15**	**P**	**7**	1626.8680	667.3105		
1754.9309	570.2577		**570.2644**	**16**	**N**	**6**	1740.9109	570.2577		
1883.9735	456.2148			**17**	**E**	**5**	1869.9535	456.2148		
1981.0262	327.1722			**18**	**P**	**4**	1967.0062	327.1722		
2038.0477	230.1194			**19**	**G**	**3**	2024.0277	230.1194		
2095.0692	173.0980			**20**	**G**	**2**	2081.0492	173.0980		
	116.0765			**21**	**L**	**1**		116.0765		

Supplementary Table 3: Primers and plasmids used in this study. 15 nt overhangs complementary to pJK301 are underlined.

Primers/Plasmids	Sequence	Application
**UP-fw**	AGCCTACACAAGCTATTAAACTTTCGTATTAAGAG	PCR amplification
**UP-rev**	CGGGCCCCCCCTCGATGATTGCTGCAATGTGGTTGGC	PCR amplification
**DOWN-fw**	ACCGCGGTGGCGGCCCAAGGAAGTGGATAAGGAAG	PCR amplification
**DOWN-rev**	GCAGCCCGGGGGATCGAAACGGTCTTTATTCCGGG	PCR amplification
**Ma5**	ATCCTACTGCGACACCCTG	Confirmation of the mutant strain and sequencing
**Ma6**	TCGGTCATGAGAATCACTCC	Confirmation of the mutant strain and sequencing
**DD15**	ATATGGAAGTAGTTGTCGACGTAG	Amplification of *ma4551*
**DD73**	CAAGGAAGTGGATAAGGAAGCCCA	Amplification of *ma4551*
**pJK301**	pBluescript-based vector containing Frt-*pac*-*hpt*-Frt construct[1]	Linear cassette for the deletion of *ma4551*

Supplementary Table 4: Strains used in this study.

Strain	Genotype	Reference
E. coli DH10B	F–, *mcrA*, Δ*(mrr-hsdRMS-mcrBC)*, Δ*lacX74*, *Φ80*, *lacZ*Δ*M15*, *recA1*, *endA1*, *araD139*, Δ*(ara,leu)*, *7697*, *galU*, *galK*, *rpsL*, *nupG*, *λ–*	[2]
M. acetivorans WWM1	C2A, Δ*hpt*	[3]
M. acetivorans Mko4551	WWM1, Δ*ma4551*::*frt-pac-hpt-frt*	This study

Supplementary Figure 1

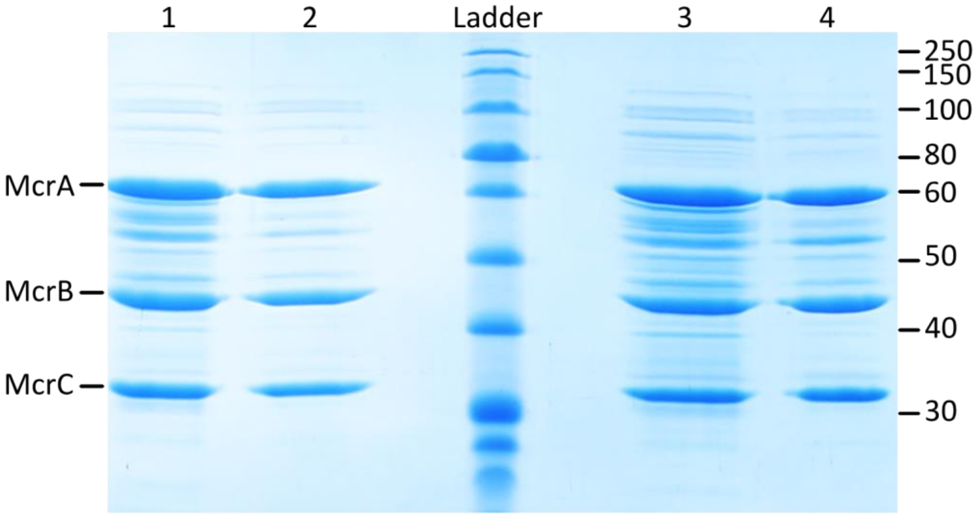

Figure 1: SDS-PAGE analysis of the methyl-coenzyme M reductase fractions after purification by anion exchange chromatography (AIEX) and size exclusion chromatography (SEC). Lane 1: pooled fractions containing MCR from *M. acetivorans* WWM1 after AIEX, lane 2: pooled fractions containing MCR from *M. acetivorans* WWM1 after AIEX and SEC, lane 3: pooled fractions containing MCR from *M. acetivorans* Mko4551 after AIEX and lane 4: pooled fractions containing MCR from *M. acetivorans* Mko4551 after AIEX and SEC. McrA: methyl-coenzyme M reductase, α subunit; McrB: methyl-coenzyme M reductase, β subunit and McrC: methyl-coenzyme M reductase, γ subunit. Ladder: Broad Range Protein Ladder (10-250 kDa, New England BioLabs).

Supplementary Figure 2

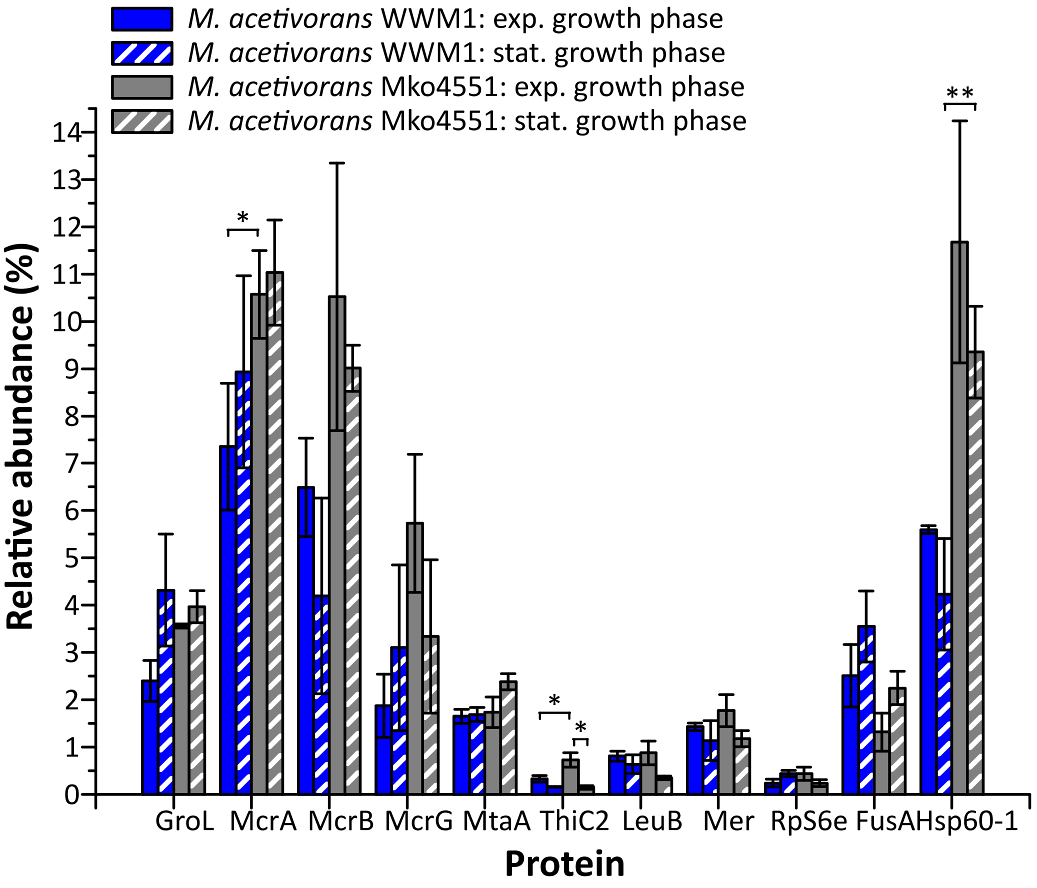

Figure 2: Label-free quantification of the most abundant proteins in *M. acetivorans* and calculation of the average protein relative abundance in relation to the overall protein content *via* nLC/HR-ESI-MS/MS. GroL: 60 kDa chaperonin; McrA: methyl-coenzyme M reductase, α subunit; McrB: methyl-coenzyme M reductase, β subunit; McrG: methyl-coenzyme M reductase, γ subunit; MtaA: methylcobamide:CoM methyltransferase isozyme M; ThiC2: phosphomethylpyrimidine synthase 2; LeuB: isocitrate/isopropylmalate dehydrogenase family protein; Mer: 5,10-methylenetetrahydromethanopterin reductase; RpS6e: 30S ribosomal protein S6e; FusA: elongation factor 2; Hsp60-1: heat shock protein 60. Paired two-sample *t*-test of three replicates and standard deviation are shown (* $p < 0.05$, ** $p < 0.01$).

Supplementary Figure 3

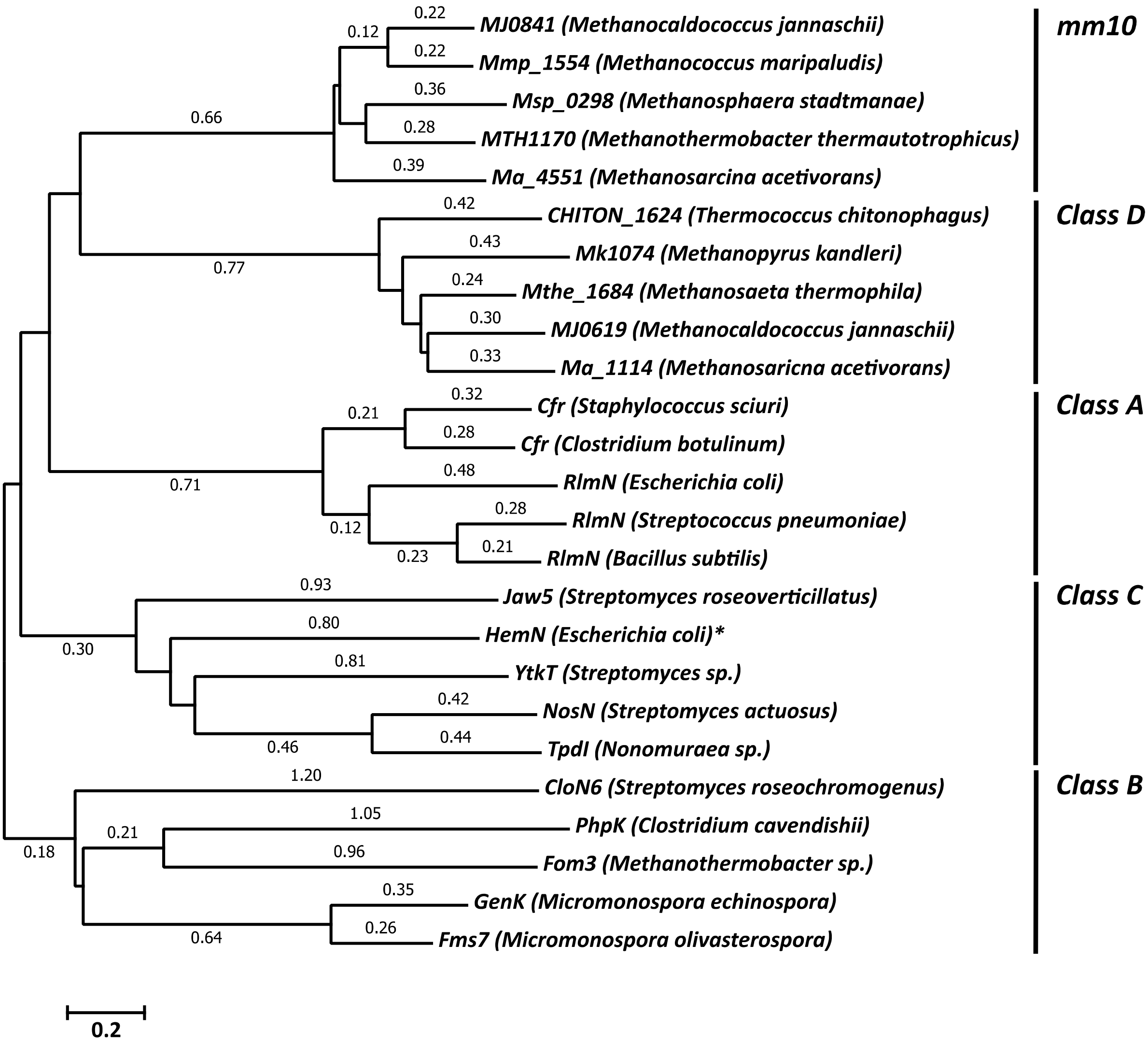

Figure 3: Phylogenetic tree of representatives of the four Radical SAM methyltransferase classes A-D and mm10. The evolutionary relationship was inferred using the Neighbor-Joining method[4]. The optimal tree with the sum of branch length = 17.1890 is shown. The tree is drawn to scale, with branch lengths (indicated above the branches) in the same units as those of the evolutionary distances used to infer the phylogenetic tree. The analysis comprised 25 amino acid sequences and was conducted in MEGA7[5]. *HemN is the Radical SAM enzyme coproporphyrinogen III dehydrogenase and not a methyltransferase. It was included in the analysis, because the Class C methyltransferases contain a C-terminal HemN-like domain.

Supplementary Figure 4

Ma_4551 (*M. acetivorans*)	---------------------------MEVVVDVGGNPGV
MJ0841 (*M. jannaschii*)	------------------------MKEANLLIDLRGEPGI
Mmp_1554 (*M. maripaludis*)	MNQKFGFNNIDAGFGFEDTDNNDSNDKSQLLIDLKGEPGK
Msp_0298 (*M. stadtmanae*)	------------------------------MADVGGIPGK
MTH1170 (*M. thermautotrophicus*	---------------------------MQIIADLGGIPGK
Ma_4551 (*M. acetivorans*)	DCKGFCKYCYFKKVK--DIQPLGCKYCLPFKKGCDYCTRS
MJ0841 (*M. jannaschii*)	NCNGFCKFCYFRKVNKNNPQPFGCRYCQ-FTVGCDYCMYS
Mmp_1554 (*M. maripaludis*)	NCGGFCKFCYFRKVNFNNPEPLGCGQCT-FQIGCDYCTNS
Msp_0298 (*M. stadtmanae*)	NCRGFCEYCYFKKVT--QKKVLGCKNCPPGQIGCPHCTT-
MTH1170 (*M. thermautotrophicus*	DCRGFCRYCYFRKVG--EFEAFGCKNCSPGRVGCETCGKG
Ma_4551 (*M. acetivorans*)	VKESYSGFKSLQMVLEETANKLYFTSGEVK--KFTVSGGG
MJ0841 (*M. jannaschii*)	VREINGDFIPLPFALMELQSSLLFKRYS----KVNLTAGG
Mmp_1554 (*M. maripaludis*)	IREINGNFIPVPFAIEQVQNAIIFKRYE----KVNITSGG
Msp_0298 (*M. stadtmanae*)	DTNMTRDYFPPYQVLSSLQTNIFQTRLP-KDTLVNITGDG
MTH1170 (*M. thermautotrophicus*	VAEIGNEFLPPFLVMGNVQTTLMMGNFQDRDVKINISGGG
Ma_4551 (*M. acetivorans*)	DLSCYPELKSLITFLSQFNTPIHLGYTSGKGFSKPDDALF
MJ0841 (*M. jannaschii*)	DVSCYPQLEELCKAINNIGLKIHLGYTSGKGFDNVEIAKN
Mmp_1554 (*M. maripaludis*)	DTSYYPYLEDLCKIINEMGLKIHLGYTSGKGLKGIESAKY
Msp_0298 (*M. stadtmanae*)	DVSCYPHLLELTKGIHDMGLPIHLGYTSGKGIDDVSTVDK
MTH1170 (*M. thermautotrophicus*	DVSCYPHLEELTAGLGEFGIPIHLGYTSGKGIDDPGIASR
Ma_4551 (*M. acetivorans*)	YIDNGVTEVSFTVFATDPALRAEYMKDPEPEAS-IQVLRD
MJ0841 (*M. jannaschii*)	LVDYGVDEVTFSVFSTNPKLRKEWMNDKNAETA-LKCLRY
Mmp_1554 (*M. maripaludis*)	LVDSGVDEVTFSVFSTDPNLRKEWMNDKNPEQS-LECLKY
Msp_0298 (*M. stadtmanae*)	LINNGVIETTYTAFSTDAKLREKWMHDPTADVS-IQALKR
MTH1170 (*M. thermautotrophicus*	LIENGVDEVTFTVFSANPDLRREWMRDENAFQGPLEALRI
Ma_4551 (*M. acetivorans*)	FCTHCEVYGAIVLLPGINDGEVLEKTLCDLENMGAKGAIL
MJ0841 (*M. jannaschii*)	FCENCEVHCAIIVIPGVNDGEELKKTVSDLVDWGANAVIL
Mmp_1554 (*M. maripaludis*)	FCENCETHCAIIVIPGVNDGEVLKNTISDLVSWGANAVIL
Msp_0298 (*M. stadtmanae*)	FCESCDVHAASIIIPGVNDGDILAKTCADLESWGAKALIL
MTH1170 (*M. thermautotrophicus*	FCESCEVHAASVIIPGVNDGDDLLETCARLEEWGATGFIM
Ma_4551 (*M. acetivorans*)	MRFANFQENGLILNNSPIIPGITPHTVSEFTEIVRSSAEK
MJ0841 (*M. jannaschii*)	MRFANSEEQGLILGNAPLIEGIKPHSVEEFKNIVDEIHNE
Mmp_1554 (*M. maripaludis*)	MRFANNVENGLIFGNEPLIEDITVQSVEEFGTLVKEMHEL
Msp_0298 (*M. stadtmanae*)	MRFANTANQGLILNNGPIIPGIKEQTLTEFENLVRETAKN
MTH1170 (*M. thermautotrophicus*	MRFANYEHQGLILGNEPIIEGVEPHTVSEFEELVRAIDRE
Ma_4551 (*M. acetivorans*)	HP-SIRITGTPLEDPLIGSPFAIR--NVPEALLKLP-RVS
MJ0841 (*M. jannaschii*)	FGDYIRVTGTPLHDPVAGTPFALAKEENSHILERLKDKIN
Mmp_1554 (*M. maripaludis*)	FGNSVRISGTPVYDPITNTPFAIYYEEE--ILDELRAKIK
Msp_0298 (*M. stadtmanae*)	YN--LRVTGTPVCDPETDAPYALSKDKNKEYLEILT-PVR
MTH1170 (*M. thermautotrophicus*	FN--LRVTGTPVCDPQNGTPFVLAADSSREYLEILP-EIQ
Ma_4551 (*M. acetivorans*)	KKATIITGQVAASRLTEIFEALGG--TVNVIPVKKDIGCL
MJ0841 (*M. jannaschii*)	GEATIITGNVAYPFLKKIFDET----SVNVVKVNKDIADL
Mmp_1554 (*M. maripaludis*)	AEATIITGNVSYIYLSKIFENT----PIKVIPVNKDIADL
Msp_0298 (*M. stadtmanae*)	AEATIVTSKISAPYIEKILENLNAADYVNVVSTSQEIACL
MTH1170 (*M. thermautotrophicus*	GEATIITGSIAAPYIRRIIHNLGADDLVNVVGVNKDIACL
Ma_4551 (*M. acetivorans*)	ITIDDFKALDLSEVTETVFIPGRAFVHDMEIKEALRRDGV
MJ0841 (*M. jannaschii*)	ITAKDLEKLDLKDVKETVFIPPKAFVHDRVAEEILRRDGV
Mmp_1554 (*M. maripaludis*)	ITKTDLETVDLSQVTDTVLYPVNALVHERDAEDIFTKDGM
Msp_0298 (*M. stadtmanae*)	ITEHDLREIDLNEVKDTVIIPGRCYVHDLVAEKIFRSDGK
MTH1170 (*M. thermautotrophicus*	ITLRDLEEVDPGDLRETVIIPGRAFVHDMQAREVLSRDGV
Ma_4551 (*M. acetivorans*)	DRIVRRGPERLSVDGEMSIGMTREEVLELEVENFTELIGQ
MJ0841 (*M. jannaschii*)	DRIVVRGVEQLTLDGEVSGIYTREEALKFEIEAFEELIGM
Mmp_1554 (*M. maripaludis*)	KRIVLRGIDKLTMDGEVSGIFTKEEAIEFEINAFNELIEK
Msp_0298 (*M. stadtmanae*)	FRLIHRGPDMLTADGEMSGTLSKNDVLKQELMAFEDLIEL
MTH1170 (*M. thermautotrophicus*	DRIVVRGPDRLTVDGEMSGTLSEYDVIEREMEAFYELIQA
Ma_4551 (*M. acetivorans*)	INSLGLPLE
MJ0841 (*M. jannaschii*)	INFFGMKKQ
Mmp_1554 (*M. maripaludis*)	INFFGNPI-
Msp_0298 (*M. stadtmanae*)	INYMGVHI-
MTH1170 (*M. thermautotrophicus*	INFFGASE-

Figure 4: Amino acid sequence alignment of different methanogenesis marker 10 proteins from distinct methanogenic archaea. Conserved amino acid residues are highlighted in color.

References

1. Welander, P. V. & Metcalf, W. W. Mutagenesis of the C1 oxidation pathway in *Methanosarcina barkeri*: new insights into the Mtr/Mer bypass pathway. *J Bacteriol* **190,** 1928–1936 (2008).
2. Durfee, T. *et al.* The complete genome sequence of *Escherichia coli* DH10B. Insights into the biology of a laboratory workhorse. *J Bacteriol* **190,** 2597–2606 (2008).
3. Pritchett, M. A., Zhang, J. K. & Metcalf, W. W. Development of a markerless genetic exchange method for *Methanosarcina acetivorans* C2A and its use in construction of new genetic tools for methanogenic Archaea. *Appl. Environ. Microbiol.* **70,** 1425–1433 (2004).
4. Saitou, N. & Nei, M. The neighbor-joining method. A new method for reconstructing phylogenetic trees. *Mol Biol Evol* **4,** 406–425 (1987).
5. Kumar, S., Stecher, G. & Tamura, K. MEGA7. Molecular evolutionary genetics analysis Version 7.0 for bigger datasets. *Mol Biol Evol* **33,** 1870–1874 (2016).
